# Acute Disseminated Encephalomyelitis: An Unusual Presentation of Human Immunodeficiency Virus Infection

**DOI:** 10.1155/2020/1020274

**Published:** 2020-06-06

**Authors:** Pedro Martínez-Ayala, Miguel Angel Valle-Murillo, Oscar Chávez-Barba, Rodolfo I. Cabrera-Silva, Luz A. González-Hernández, Fernando Amador-Lara, Moises Ramos-Solano, Sergio Zúñiga-Quiñones, Vida Verónica Ruíz-Herrera, Jaime F. Andrade-Villanueva

**Affiliations:** ^1^HIV Unit Department, University Hospital “Fray Antonio Alcalde”, University of Guadalajara, Guadalajara, JAL, Mexico; ^2^Neurology Department, Centro de Especialidades Médicas Del Sureste, Mérida, Yucatán, Mexico; ^3^Radiology Department, University Hospital “Fray Antonio Alcalde”, University of Guadalajara, Guadalajara, JAL, Mexico; ^4^HIV and Immunodeficiencies Research Institute, Clinical Medicine Department, CUCS-University of Guadalajara, Guadalajara, JAL, Mexico

## Abstract

**Background:**

Acute disseminated encephalomyelitis (ADEM) is a rare inflammatory and demyelinating disorder of the central nervous system, with a distinct tendency to a perivenous localization of pathological changes. Children are the most affected population and frequently presented after exanthematous viral infections or vaccination. Due to the rarity of this disease, the annual incidence rate in the population is not precisely known. *Case Presentation*. Here, we present a 28-year-old male HIV-1 positive patient with an acute confusional state, a diminished alert status characterized by somnolence, hypoprosexia, and complex visual hallucinations. Neuroimages reported white matter demyelinating lesions, mainly affecting the semioval centers, the frontal lobe, and the left parietal lobe; hypointense on T1-weighted images, hyperintense on T2-weighted images and fluid-attenuated inversion recovery weighted images, DWI with restricted diffusion, and a parietal ring-enhancing lesion after IV gadolinium administration. *Discussion*. In HIV positive patients, the demyelinating disorders have a broader clinical spectrum that could be explained by the immunosuppressed state of the patients, the evolution of the disease, the use of medications, the opportunistic infections, and the environment. Due to this highly variable clinical spectrum, ADEM is a significant challenge for the physicians in HIV positive patients, causing a delay in the diagnosis and treatment.

**Conclusion:**

We suggest that ADEM should be considered among the differential diagnosis in HIV-infected patients with focal or multifocal neurological symptoms, particularly in encephalopathies with multifocal central nervous system involvement without severe immunosuppression.

## 1. Introduction

Acute disseminated encephalomyelitis (ADEM) is a rare inflammatory and demyelinating disorder (DD) of the central nervous system (CNS). Distinctively, ADEM's pathological changes tend for a perivenous localization [1]. Children are the most affected population (mainly younger than 15 years), frequently presented after exanthematous viral infections or vaccination. Other well-documented associations include HIV, influenza virus, Epstein–Barr virus, Herpes Simplex virus, or Cytomegalovirus infection and postsurgical interventions [2]. Due to its rarity, ADEM's annual incidence in the population is unknown. A study from 1991 to 2000, in 3 pediatric hospitals from San Diego, California, reported an incidence of 0.4 per 100,000 people-years in persons less than 20 years of age [1]. ADEM is generally self-limited and monophasic, with clinical remission expected within four weeks [3].

In HIV patients, ADEM develops as a multifocal disorder of the CNS, becoming monophasic during seroconversion, even when the immune system remains competent. However, a study of seven HIV-1 positive patients with ADEM reported an increased frequency of atypical presentations [4, 5].

ADEM's pathogenesis has an autoimmune origin, either by molecular mimicry (epitopes with structural homology to myelin proteins) or activation of pre-existent T-cells with antimyelin activity. Regardless, they cause a demyelinating process and perivenular inflammation [6]. The diagnosis relies on clinical and radiological findings; Table 1 shows the criteria for ADEM [7, 8].

Magnetic Resonance Imaging (MRI) findings include large brain lesions of at least 2 cm, either disseminated or confluent, and they can involve the white matter, cortex, and deep grey nuclei. The lesions are generally multiple, but large unique lesions can also affect both hemispheres; plus, the involvement of the deep grey matter helps to distinguish ADEM from multiple sclerosis (MS). Lesions are hypointense on T1-weighted images and hyperintense in T2-weighted images and short TI inversion recovery (STIR) weighted sequences. However, lesions with intense gliosis can be observed hyperintense in T1-weighted images. In diffusion-weighted magnetic resonance imaging (DWI), restriction, nodular lesions, and ring enhancement are standard features after intravenous (IV) contrast injection [9].

At the spinal cord level, the radiological findings include focal lesions in the craniocervical junction and longitudinally extensive lesions affecting at least three intervertebral spaces [9].

In this case report, we present a 28-year-old male HIV-1 positive patient with clinical, imaging, serological, and cerebrospinal fluid (CSF) findings consistent with ADEM.

## 2. Case Presentation

A 28-year-old male patient admitted to the emergency department presented a tonic-clonic seizure, left arm paresis, paraparesis, two months of gait disturbance, and fever (38°C). The patient was diagnosed with HIV-1 three months ago. His CD4^+^ T-cell count was 669 cells/*μ*L, with a viral load of 23,800 c/mL, CDC stage A1, and naïve to antiretroviral therapy (ART). The patient presented a confusional state, somnolence, hypoprosexia, and complex visual hallucinations. The left arm's strength was diminished (3/5), and the lower limbs had symmetric paresis (2/5) and symmetrically augmented myotatic reflexes. Babinski's plantar reflex was absent. The rest of the neurological examination: cranial nerves, optic fundus, and papilla did not have alterations, sensory examination was normal, and there were no meningeal signs, abnormal movements, or ataxia.

**Figure 1:**
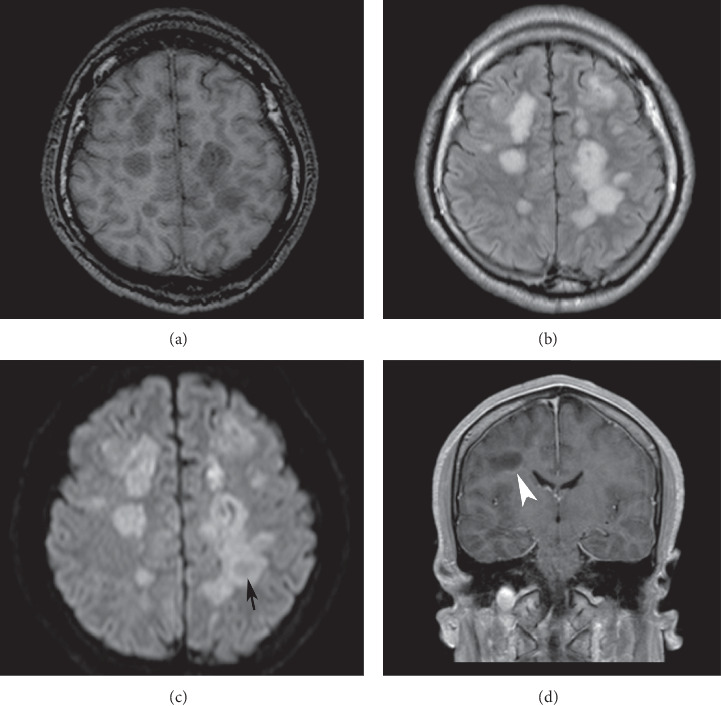
Case report's neuroimages. Axial and coronal MRI with T1 (a), FLAIR (b), diffusion (c), and contrast-enhanced T1, (d) weighted images show various predominant white matter (nodular and confluent) lesions hypointense on T1, hyperintense on T2 (not shown), and FLAIR. With diffusion restriction, some of them had fewer signals at the center (black arrow). After gadolinium administration, most of the lesions have mild incomplete annular enhancement (white arrowhead). MRI : magnetic resonance imaging; FLAIR : fluid-attenuated inversion recovery.

The CSF was clear and had the following findings: proteins 27 mg/dl, glucose 74 mg/dl (serum glucose 90 mg/dl), and a white blood cell count of 20 cells/mm^3^ (54% lymphocytes) with no evidence of malignant cells nor oligoclonal bands. The cryptococcus antigen test, polymerase chain reaction (PCR) for *M. tuberculosis*, VDRL, bacterial cultures, and Gram and Ziehl Neelsen stains in CSF were negative. Serum IgG serology for Toxoplasma Gondii was negative. MRI reported white matter demyelinating lesions, mainly affecting the centrum semiovale, the frontal lobe, and the left parietal lobe; hypointense on *T1*-weighted images, hyperintense on *T2*-weighted images and fluid-attenuated inversion recovery (FLAIR) weighted images, DWI with restricted diffusion, and a parietal ring-enhancing lesion after IV gadolinium administration (Figure 1).

After meningeal cryptococcal and meningeal tuberculosis infection was discarded, we started ART with abacavir/lamivudine/dolutegravir. Since, ART initiated after symptoms onset, immune reconstitution inflammatory syndrome (IRIS) criteria are not met, we started ART with Abacavir/Lamivudine/Dolutegravir. By exclusion of other diagnoses, ADEM was diagnosed, and the patient received high doses of IV methylprednisolone (1 g/day) for five consecutive days; subsequently, he showed neurological function improvement. After three years of follow-up, the patient showed complete neurological remission with no relapses. He continued on ART with good adherence and undetectable.

## 3. Discussion

This report describes a recently diagnosed HIV-infected male with a CD4^+^ T-cell count of 669 cells/*μ*L and a sub-acute monophasic course characterized by fever, encephalopathy, and multifocal neurological deficits. Opportunistic infections were unlikely due to his relatively preserved immune status (CD4^+^ T-cell count was 669 cells/µL); regardless, opportunistic infections were considered and discarded. MRI showed confluent white matter lesions, and the patient had an excellent response to steroid therapy. We established a diagnosis of ADEM after fulfillment of the “ADEM 2012 criteria from the International Pediatric multiple sclerosis study group”; it states the patient should have a polyfocal clinical CNS event. In our case, the subject showed multiple signs corresponding to polyfocal involvement of the CNS: triparesis, convulsions, and encephalopathy, this last one being an essential element if fever was not the cause. MRI was compatible with confluent demyelinating lesions involving white matter and deep grey matter both findings are highly suggestive of ADEM lesions. [7].

Because of the high cost in our country, we were not able to perform antibody tests such as anti-acuaporin 4 antibody (AQP4) and myelin oligodendrocyte glycoprotein antibodies (anti-MOG). However, the clinical picture was not suggestive of opticospinal demyelinating syndrome and the anti-MOG serostatus in the context of ADEM is clinically helpful to establish risk of recurrence but not necessary for a diagnosis.

In the context of an HIV-infected patient, the differential diagnosis for fever and encephalopathy becomes more complicated.  Table 2 summarizes the clinical and imaging characteristics for the principal differential diagnosis. All these pathologies usually manifest a focal or multifocal neurological syndrome suggesting a space-occupying lesion, except for cryptococcosis, which shows an intracranial hypertension syndrome and sub-acute meningitis [10].

The typical course of ADEM is a neurological event with an acute or sub-acute establishment with a monophasic progression. Signs and symptoms are multifocal, including encephalopathy, which suggests multiple lesions that involve the ascending reticular activating system. Commonly, it appears with alertness deterioration and even a state of coma. Like other DD, it can affect the rest of CNS like optic nerves and spinal cord. In neuroimaging, multiple supra- and infra-tentorial lesions, predominant in the white matter, can be observed, frequently affecting the cortex and the grey nuclei at the basal ganglia and brain stem. In adults, ADEM differential diagnosis is broad, as it is less frequent in adulthood than in childhood. Similarly, the acute DD forms, such as Marburg, Hurst, or Balo disease, could arise to a neurological condition of acute onset with multifocal symptoms and single or multiple demyelinating lesions in neuroimages. Also, an isolated clinical syndrome for the first MS episode is presented as a unifocal or multifocal episode and with demyelinating lesions in the brain and the spinal cord. Neuromyelitis optica presents extensive myelitis that can be accompanied by optic neuritis and, rarely, by supratentorial lesions, which can help clinically to distinguish it from ADEM [9].

## 4. Conclusions

We consider that ADEM, more than a disease, represents a group of pathologies that have demyelinating lesions in the CNS and immunological dysfunction as a common pathway. In HIV, a potential mechanism is the generation of incomplete reverse transcripts (HIV's DNA), which stimulate an intense inflammatory response and a subsequently CD4+ T-cell depletion, through damage to the stem cells and the thymus [11]. Thus, the presence of “autoreactive” circulating T-cells predisposes the development of ADEM. Particularly in HIV-infected patients, the DD clinical spectrum becomes reasonably broad, possibly explained by the immunosuppressed state of the patients, the evolution of the disease, the use of medications, opportunistic infections, and the environment. Due to this diverse clinical spectrum, ADEM is a challenge for physicians, delaying the diagnosis and treatment. ADEM's treatment includes high IV corticosteroid dosage, immunoglobulin, or plasmapheresis, which accelerate the patient's recovery and reduce the number of active lesions [12, 13].

In conclusion, we suggest that ADEM should be considered as a differential diagnosis in an HIV-infected patient presenting focal or multifocal neurological symptoms, particularly in patients with encephalopathy and without severe immunosuppression (CD4^+^ T-cell count >200 cells/*μ*L) or when neuroimages show focal lesions.

## Figures and Tables

**Table 1:** ADEM 2012 criteria from the International Pediatric Multiple Sclerosis Study Group.

ADEM is divided into three groups	
Monophasic ADEM	(i) A first polyfocal clinical neurological event with a presumed inflammatory cause
	(ii) A polysymptomatic clinical picture that includes encephalopathy
	(iii) Absence of new/recent signs and symptoms or MRI findings after three months of ADEM diagnosis

Multiphasic ADEM	(iv) A new ADEM event three months or more after the initial episode that involves unaffected areas from the previous event
	(v) It can be associated with novel clinical and MRI findings or to previously documented findings
	(vi) It must take place within one month after completing steroid treatment

Recurrent ADEM	(vii) Recurrence of the initial signs and symptoms within three months or more after the initial episode
	(viii) Absence of new lesions based on medical history, physical examination, and neuroimaging
	(ix) MRI without new lesions; however, previous lesions can be increased in volume

**Table 2:** Clinical and image comparison with the primary differential diagnoses for ADEM.

Clinical entity	Clinical findings	Neuroimaging	Comments
ADEM	Monophasic clinical picture. Encephalopathy + focal symptoms + myelopathy	Multiple lesions in WM and cortex or deep grey nuclei	Suspect in the light of compatible clinical picture, responds well to IV steroids
MS	Insidious clinical picture with clinical relapses. Rarely with encephalopathy	Lesions in WM, rarely affecting cortex Lesions in different ages	Absence of encephalopathy and frequent relapses distinguish it from ADEM
NMO	It affects only the optic nerves and the spinal cord. Patients do not get encephalopathy	Produces widely extensive myelitis, and it is not inclined to affect supratentorial regions. Optic nerve involvement	Absence of encephalopathy, differentiate from ADEM, and highly aggressive progression
TOXOPLASMA ENCEPHALITIS	Progressive sub-acute clinical picture, focal clinic + encephalopathy. Not accompanied by myelopathy	Lesions in basal grey nuclei and the cortico-subcortical junction	Most common cause of focal neurologic syndrome in HIV
PML	Cognitive impairment prevails + focal signs; visuals are common	Diffuse lesions; it affects mostly U fibers and parieto-occipital regions. Contrast-enhanced +	Patients do not respond to immunotherapy. Suspect if low CD4 cell count
PCNSL	Progressive sub-acute clinical picture, focal clinic + encephalopathy	Closely similar to toxoplasma encephalitis, lesions in the corpus callosum, and periventricular enhancement	Responds initially to steroids. It requires SPECT, PET, and biopsy
HIV-ASSOCIATED DEMENTIA COMPLEX	Progressive cognitive deterioration + gait disturbance	Diffuse lesions restricted to U fibers; no contrast-enhanced	Disease with a long evolution and a progressive course

ADEM : acute disseminated encephalomyelitis; MS : multiple sclerosis; NMO : neuromyelitis optica; PML : progressive multifocal leukoencephalopathy; PCNSL : primary central nervous system lymphoma; HIV : human immunodeficiency virus; WM : white matter; IV : Intravenous; SPECT : single photon emission computed tomography; PET : positron emission tomography.

## Data Availability

The authors confirm that the data supporting the findings of this study are available within the article.
